# Maslinic acid alleviates intervertebral disc degeneration by inhibiting the PI3K/AKT and NF-κB signaling pathways: Role of MA in alleviating intervertebral disc degeneration

**DOI:** 10.3724/abbs.2024027

**Published:** 2024-03-18

**Authors:** Yichen Que, Chipiu Wong, Jincheng Qiu, Wenjie Gao, Youxi Lin, Hang Zhou, Bo Gao, Pengfei Li, Zhihuai Deng, Huihong Shi, Wenjun Hu, Song Liu, Yan Peng, Peiqiang Su, Caixia Xu, Anjing Liang, Xianjian Qiu, Dongsheng Huang

**Affiliations:** 1 Department of Orthopedic Surgery Sun Yat-sen Memorial Hospital of Sun Yat-sen University Guangzhou 510120 China; 2 Panyu Hospital of Chinese Medicine Department of Minimally Invasive Spine Surgery Guangzhou 511408 China; 3 Department of Orthopedic Surgery the First Affiliated Hospital of Sun Yat-sen University Guangzhou 510080 China; 4 Research Centre for Translational Medicine the First Affiliated Hospital of Sun Yat-sen University Guangzhou 510006 China

**Keywords:** maslinic acid, intervertebral disc degeneration, NF-κB, PI3K, senescence

## Abstract

Intervertebral disc degeneration (IDD) is the cause of low back pain (LBP), and recent research has suggested that inflammatory cytokines play a significant role in this process. Maslinic acid (MA), a natural compound found in olive plants ( *Olea europaea*), has anti-inflammatory properties, but its potential for treating IDD is unclear. The current study aims to investigate the effects of MA on TNFα-induced IDD *in vitro* and in other *in vivo* models. Our findings suggest that MA ameliorates the imbalance of the extracellular matrix (ECM) and mitigates senescence by upregulating aggrecan and collagen II levels as well as downregulating MMP and ADAMTS levels in nucleus pulposus cells (NPCs). It can also impede the progression of IDD in rats. We further find that MA significantly affects the PI3K/AKT and NF-κB pathways in TNFα-induced NPCs determined by RNA-seq and experimental verification, while the AKT agonist Sc-79 eliminates these signaling cascades. Furthermore, molecular docking simulation shows that MA directly binds to PI3K. Dysfunction of the PI3K/AKT pathway and ECM metabolism has also been confirmed in clinical specimens of degenerated nucleus pulposus. This study demonstrates that MA may hold promise as a therapeutic agent for alleviating ECM metabolism disorders and senescence to treat IDD.

## Introduction

Low back pain (LBP) is a prevalent condition in modern population [1]. The primary etiology of this pain is intervertebral disc degeneration (IDD) [2], which is a complex condition that requires further research to understand its mechanism progression [3]. The intervertebral disc (IVD) contains three parts: annulus fibrosus, nucleus pulposus (NP) and cartilaginous endplate. NPs include specialized cells called NPCs that synthesize and secret extracellular matrix (ECM). IDD is primarily caused by functional deterioration of the NP [4].

The process of IVD tissue degeneration in adulthood is caused by multiple factors, including incorrect biomechanics, inflammatory factors such as tumor necrosis factor α (TNFα), and the senescence of NPCs [4]. NPCs are responsible for maintaining balance within the IVD environment by regulating ECM metabolism. However, metabolic disorders of the ECM can occur due to insufficient anabolism and increased catabolism, which leads to the downregulation of COL2A1 and ACAN levels, as well as the upregulation of the expressions of MMPs and ADAMTs [5].

A previous study showed that TNFα is closely linked to IDD [6]. When the TNFα concentration is elevated, the secretion of MMPs and ADAMTSs increases, while that of COL2A1 decreases [5]. This reaction speeds up the dysregulation of ECM metabolism in the NP, causing cellular senescence and worsening the inflammatory microenvironment [7]. Additionally, TNFα can increase the expressions of various proinflammatory cytokines, further accelerating the progression of IDD [8]. Moreover, TNFα has been implicated in nerve irritation and growth [9]. In summary, TNFα plays a crucial role in the development of IDD. Therefore, therapies targeting TNFα are promising for preventing IDD and LBP in the future.

The NF-κB pathway is essential for the TNFα-induced inflammatory response and IDD development [10]. When stimulated by different factors, such as TNFα, IKK-alpha/beta and transforming growth factor-β-activated kinase 1 (TAK1) phosphorylate IκBα. This leads to the production of nuclear localization signals, which causes p65 to transit to the nucleus and subsequently activate the transcription of particular genes [11]. The NF-κB pathway is involved in ECM metabolism disorders, apoptosis and senescence in NPCs [12]. The PI3K/AKT pathway regulates NF-κB through phosphorylation of p65 and IκBα [13]. In the development of IDD, NF-κB is located downstream of the PI3K/AKT pathway [14].

Several reports on reversing IDD have been published, such as related studies on exosomes [ 15‒ 17] and some examples of new classical drugs, including metformin [18] and dexamethasone [19]. However, at present, the main treatment for IDD is still limited to relieving symptoms, which cannot solve the fundamental problem of disc degeneration [20]. Currently, natural compounds and their derivatives are frequently utilized as innovative drugs for various diseases. Maslinic acid (MA), a pentacyclic triterpene compound extracted from olive *(Olea europaea)* [ 21, 22], has shown potential benefits, such as antitumor, anti-inflammatory and antioxidant functions [ 23‒ 25]. Several molecules with similar functions, such as CBX4 [26] and 17 beta-estradiol, also have the potential to protect against IVD [27]. However, the role of MA in IDD needs further evaluation.

In the present study, we investigated the effects of MA on the TNFα-induced inflammatory response and aging phenotype in NPC cells, verified these effects *in vivo*, and explored the underlying mechanism. In addition, we explored the effect of MA on PI3K. Our results showed that MA could ameliorate ECM metabolism disorders, alleviate TNFα-induced senescence in NPCs and ameliorate the progression of acupuncture-induced IDD in a rat model by modulating the PI3K/AKT and NF-κB signaling pathways. This study provides a theoretical basis for the use of MA as a therapeutic drug for IDD.

## Materials and Methods

### Antibodies and reagents

MA (molecular formula, C _30_H _48_O _4_; CAS#: 4373-41-5) was purchased from MCE (Monmouth Junction, USA), and the purity of MA exceeded 98%, as determined by HPLC. Recombinant human TNFα was purchased from R&D Systems (Minneapolis, USA). Sc-79 was purchased from Selleck (Houston, USA). A Cell Counting Kit-8 (CCK-8) was procured from Glpbio (Tianjin, China).

Antibodies against GAPDH (ab8245), COL2A1 (ab34712), ADAMTS5 (ab41037), ADAMTS4 (ab185722), MMP3 (ab52915), TNFα (ab6671), p21 (ab109520) and p16 (ab51243) were obtained from Abcam (Cambridge, UK). Antibodies against ACAN (DF7561) and p-PI3K (AF3242) were obtained from Affinity Biosciences (Changzhou, China). Antibodies against MMP9 (13667T), NF-κB p65 (6956S), p-NF-κB p65 (3033S), PI3K (4257T), AKT (4685S), p-AKT (13038T), IκBα (9242), p-IκBα (2859p), IKK (61294), p-IKK (2078) and secondary antibodies were obtained from Cell Signaling Technology (Boston, USA). An antibody against MMP13 (18165-1-AP) was obtained from the Proteintech Group (Chicago, USA).

### Tissue samples

In this study, three normal and three degenerative specimens of human NP tissues were collected. Degenerative NP tissue was taken from patients who underwent spinal surgery for disc herniation, and control tissue was collected from patients with idiopathic scoliosis without any evidence of disc degeneration. All surgeries were carried out between January 2021 and December 2022 at Sun Yat-Sen Memorial Hospital of Sun Yat-Sen University. The severity of the intervertebral disc was assessed using Pfirrmann’s grading system [28]. Patient details are shown in Table 1. This study was approved by the Medical Ethics Committee of Sun Yat-Sen Memorial Hospital (No. SYSKY-2023-934-01).

**Table:** **Table 1** Basic information of human nucleus pulposus specimens

Case	Age	Gender	Affected IVD	Pfirrmann grade
1	15	Female	L1/2	I
2	9	Male	T10/11	I
3	15	Female	L3/4	II
4	68	Female	L4/5	IV
5	62	Male	L4/5	IV
6	63	Female	L5/S1	V

L: lumbar, T: thoracic, S: sacral.

### Immunohistochemistry analysis

Tissue specimens were fixed, decalcified, dehydrated and embedded before being sectioned to a thickness of approximately 5 μm. The sections were then dewaxed, rehydrated and incubated with 3% peroxidase. After being blocked with bovine serum albumin (BSA) at room temperature for 30 min, the specimens were incubated with primary antibodies against COL2A1 (1:100), TNFα (1:100), p21 (1:100), ACAN (1:100), MMP13 (1:100), p-PI3K (1:100), MMP9 (1:100), NF-κB p65 (1:250) and p16 (1:250) at 4°C for overnight. After incubation with the corresponding HRP-conjugated secondary antibody at room temperature for 60 min, a DAB Horseradish Peroxidase Color Development kit (Beyotime, Shanghai, China) was used for detection. Images were taken with an Olympus BX63 microscope (Tokyo, Japan).

### Cell culture

NPCs were purchased from ScienCell (Carlsbad, USA) and cultured at 37°C in an incubator with 5% CO _2_ in NP cell medium (ScienCell, San Diego, USA). The NPCs were cultured at a density of approximately 1.0×10 ^6^ cells/mL and passaged for 4‒5 generations in 8 mL of medium. The NPCs were subsequently exposed to varying concentrations of MA (2.5 and 5 μM), TNFα (10 ng/mL) and Sc-79 (20 μM).

### Immunofluorescence staining

NPCs were exposed to various agents at 37°C for a duration of 48 h. The cells were incubated with antibodies against COL2A1 (1:100), ADAMTS4 (1:100), ACAN (1:100), MMP13 (1:100), NF-κB (1:100) and p65 (1:100) at 4°C for overnight, and then with the corresponding secondary antibodies (1:100) at room temperature for 60 min. Subsequently, NPCs were stained with DAPI (Solarbio, Beijing, China), and images were taken with an Olympus BX63 fluorescence microscope.

### Cell viability assay

NPC cells were seeded in 96-well plates and then treated with various concentrations of MA for different time. At the designated time points, 100 μL of fresh medium supplemented with 10 μL of CCK-8 (Glpbio) was added, and then the absorbance was measured at 450 nm using a microplate reader.

### RT-qPCR

Total RNA was extracted from cells using Trizol (Invitrogen, Carlsbad, USA). cDNA was synthesized using UnionScript First-strand cDNA Synthesis Mix for qPCR (with dsDNase) (SR511; Genesand, Guangzhou, China) according to the manufacturer’s instructions. RT-qPCR was performed on CFX 96 Real-Time PCR System (Bio-Rad, Hercules, USA) using GS AntiQ qPCR SYBR Green Master Mix (SQ412; Genesand). The expression of the *GAPDH* gene was used as a reference. To obtain the *ΔCt* values, the *Ct* value of *GAPDH* was subtracted from that of the target gene. The average *ΔCt* value was determined based on three independent experiments, and the 2 ^‒ΔΔCt^ method was used to calculate the relative expression level of each gene. The primer sequences are listed in Table 2.

**Table:** **Table 2** Sequences of primers for real-time quantitative PCR

Gene	Primer sequence (5′→3′)
*COL2A1*	F: TGGACGCCATGAAGGTTTTCT
	R: TGGGAGCCAGATTGTCATCTC
*ACAN*	F: GTGCCTATCAGGACAAGGTCT
	R: GATGCCTTTCACCACGACTTC
*ADAMTS5*	F: GAACATCGACCAACTCTACTCCG
	R: CAATGCCCACCGAACCATCT
*ADAMTS4*	F: GAGGAGGAGATCGTGTTTCCA
	R: CCAGCTCTAGTAGCAGCGTC
*MMP13*	F: TCCTGATGTGGGTGAATACAATG
	R: GCCATCGTGAAGTCTGGTAAAAT
*MMP9*	F: GGGACGCAGACATCGTCATC
	R: TCGTCATCGTCGAAATGGGC
*GAPDH*	F: GGAGCGAGATCCCTCCAAAAT
	R: GGCTGTTGTCATACTTCTCATGG

### Western blot analysis

After cell lysis, total protein was extracted using RIPA buffer. The protein concentration was determined using a BCA Protein assay kit (Biotool, Houston, USA). Thirty micrograms of protein was separated via SDS-PAGE (Solarbio) and transferred onto polyvinylidene fluoride (PVDF) membranes. Then, the membranes were blocked and incubated with primary antibodies against COL2A1, ADAMTS4, MMP3, p21, p16 (1:1000), ADAMTS5 (1:250), ACAN, p-PI3K, MMP13, NF-κB p65, p-NF-κB p65, Akt, p-AKT, PI3K, IκBα, p-IκBα, IKK, p-IKK, MMP9 (1:1000) or GAPDH (1:3000) at 4°C for overnight, followed by incubation with the corresponding HRP-conjugated secondary antibodies (1:3000) at room temperature for 60 min. After extensive wash, the protein bands were detected using an Enhanced Chemiluminescence (ECL) Detection kit (Beyotime).

### SA-β-gal staining

The NPCs were fixed following the manufacturer’s protocol for β-gal staining, and then incubated with the β-gal staining solution (Beyotime) at 37°C overnight without CO _2_. Images were taken using an inverted microscope, the number of cells was counted, and the proportion of β-gal ^+^ cells was calculated.

### Rat model of acupuncture-induced intervertebral disc degeneration

Female Sprague-Dawley rats (250‒300 g, 12 weeks, *n*=15) were purchased from Charles River Laboratory (Beijing, China), and randomly assigned into three groups: the control group, the IDD group or the IDD+MA group. Needs (21G) were used for acupuncture of the annulus fibrosus in the IDD and IDD+MA groups at a depth of approximately 5 mm in the caudal disc at Co7/8, rotated for 360° and held for 1 min. Saline was administered to rats in both the control and IDD groups on a daily basis via intragastric administration immediately after surgery, whereas those in the IDD+MA group received 100 mg/kg MA (dissolved in saline as a suspension) every day for four weeks. The animal experiment was approved by the Institutional Animal Care and Use Committee of Sun Yat-sen University (No. SYSU-IACUC-2023-001136).

### Magnetic resonance imaging (MRI)

The rats were subjected to MRI four weeks later. The rats were anaesthetized and the discs of their coccyx were detected with the Philips Intera Achieva 3.0 MR Imaging System (Amsterdam, the Netherlands), which produced sagittal T2-weighted spin-echo sequence images. The IDD severity was assessed on the basis of MRI images using the Pfirrmann grading system [28].

### Histological analysis

The tails of the rats were fixed, decalcified, dehydrated and embedded before being sectioned to an approximate thickness of 5 μm at four weeks postoperation. The sections were subjected to dewaxing, rehydration and safranin O (SO) staining as well as Hematoxylin-Eosin (H&E) staining using standard protocols. Cellular and morphological images of the intervertebral disc were obtained using a Leica DMI4000B microscope (Wetzlar, Germany).

### Molecular docking

AutoDock 4.2 software was used to generate the 3D structure of the MA molecules. The data of PI3K (PDB ID: 1E8Z) were obtained from the RCSB Protein Data Bank ( https://www.rcsb.org/), and the structure was constructed by eliminating ligand residues, water molecules and ions while introducing hydrogen atoms to enhance its stability.

The semiflexible docking method was used to dock MA with the PI3K protein. This allowed flexibility in the bond angles of MA while maintaining rigidity in PI3K. The results from molecular docking were obtained based on CDOCKER energy scores, interaction sites and types of interaction forces [29].

### Transcriptome sequencing and bioinformatics analysis

The TNFα-treated group and the TNFα with MA-treated group NPCs were both subjected to genome-wide transcriptional sequencing.

NPCs were exposed to TNFα (10 ng/mL) and TNFα (10 ng/mL) with MA (5 μM) at 37°C for a duration of 48 h and collected the total mRNA of each group. Genome-wide transcriptional sequencing was performed by Annoroad Gene Technology (Beijing, China) to determine RNA expression levels. The DESeq2 R package (version 1.16.1) was used to perform differential expression analysis of the two groups, each with three biological replicates per condition.

DESeq2 consists of statistical methods based on a negative binomial distribution to determine differential expression in digital gene expression data. The ClusterProfiler R package was utilized to conduct GO enrichment analysis on differentially expressed genes. Kyoto Encyclopedia of Genes and Genomes (KEGG) is a resource for comprehending the high-level functions and utilities of biological systems through molecular-level information analysis ( http://www.genome.jp/kegg/). Significantly enriched genes within the KEGG pathways were assessed with the clusterProfiler R package.

### Statistical analysis

Data are presented as the mean±standard deviation (SD) from a minimum of three independent experiments. Statistical analysis was performed using GraphPad Prism (GraphPad Software, La Jolla, USA). *P*<0.05 was considered statistically significant.

## Results

### Cytotoxicity of MA to NPCs

**Figure 1:**
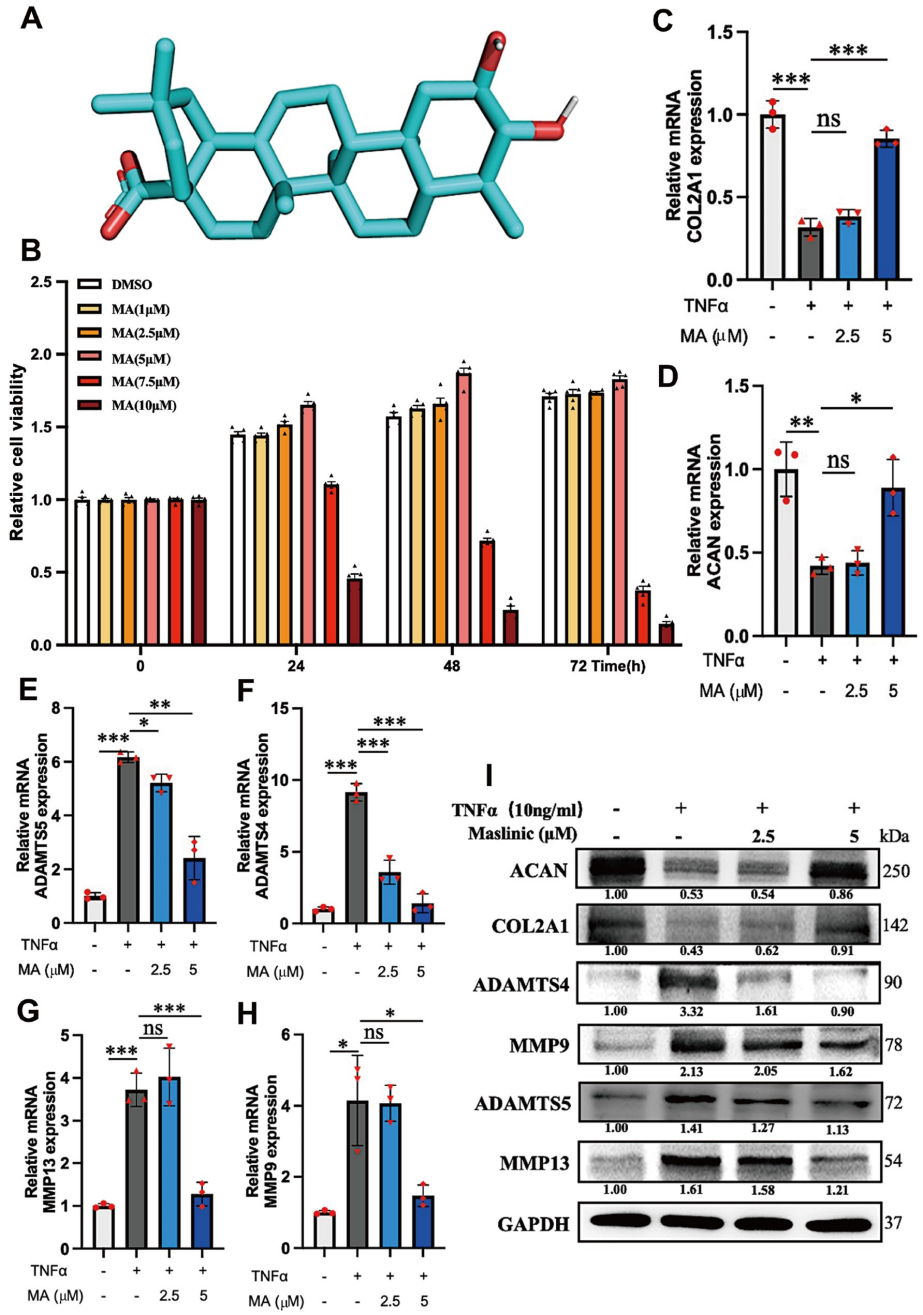
Figure 1 The impact of MA on ECM anabolism and catabolism in TNFα-treated NPCs (A) Chemical architecture of MA. (B) The cytotoxicity of MA to NPCs was evaluated at designated time points by CCK-8 assay at various concentrations. (C–H) The relative mRNA levels of COL2A1, ACAN, ADAMTS5, ADAMTS4, MMP13 and MMP9 were quantified via qPCR. (I) The protein expression levels of ACAN, COL2A1, ADAMTS4, MMP9, ADAMTS5 and MMP13 were assessed in NPCs treated with or without MA in the presence of TNFα. *P<0.05, **P<0.01, ***P<0.001.

The structure of MA is shown in Figure 1A. Previous study has shown the significant role of MA in chondrocytes at concentrations ranging from 5 to 10 μM [25]. However, its potential cytotoxic effects on NPCs have not been explored. To evaluate the toxicity of MA, NPCs were exposed to a gradient of MA concentrations (0, 1, 2.5, 5, 7.5 and 10 μM) for various durations (0, 24, 48, and 72 h), after which CCK-8 assay was used to evaluate cell viability. Our findings indicated that there was a significant decrease in cell viability at an MA concentration of 7.5 μM ( Figure 1B). Therefore, MA concentrations of 2.5 and 5 μM were selected for subsequent experiments.

### Effects of MA on the ECM of NPCs under TNFα-induced inflammation

**Figure 2:**
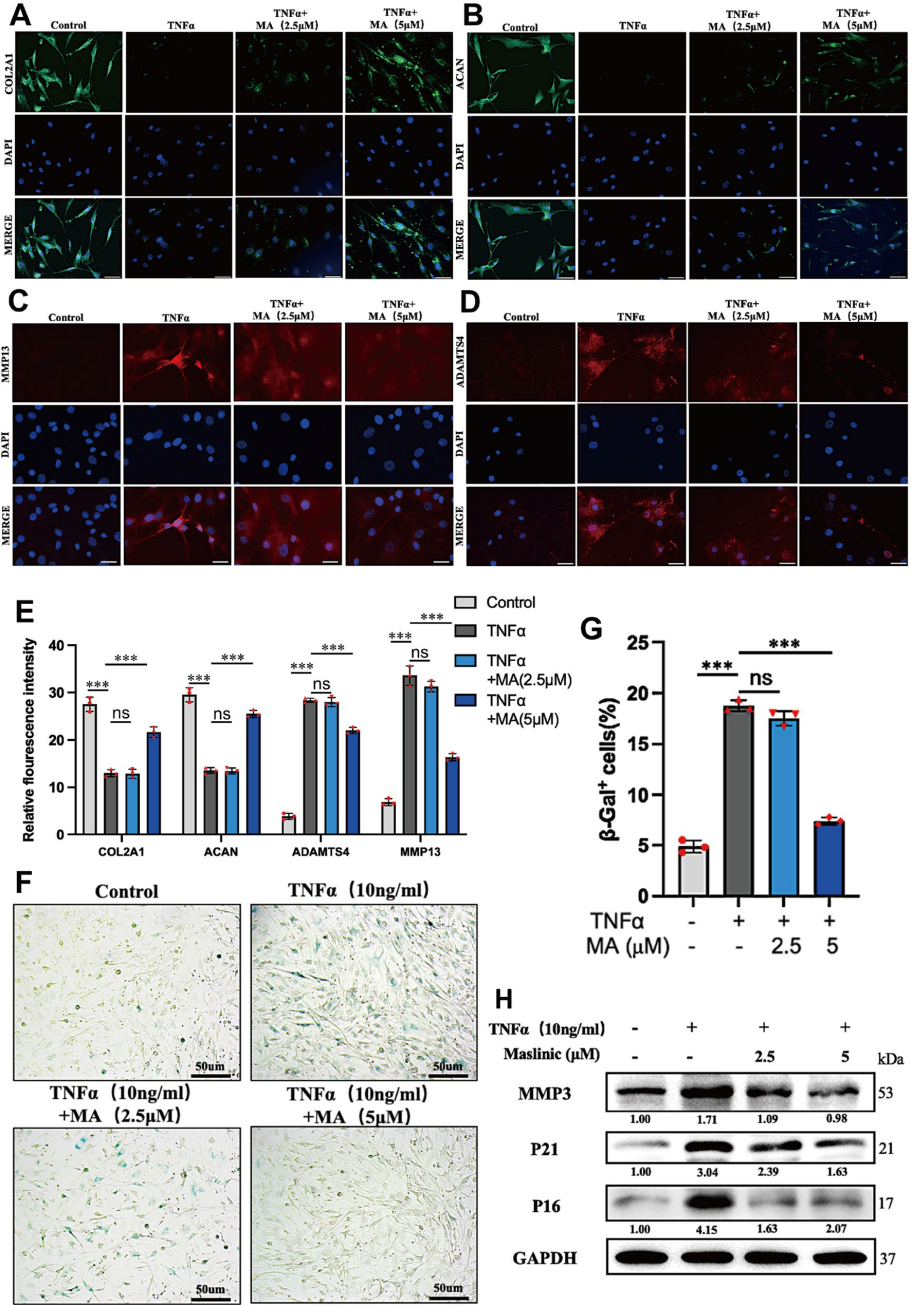
Figure 2 MA mitigated TNFα-induced senescence in NPCs (A‒D) The expressions of COL2A1, ACAN, MMP13, and ADAMTS4 were detected through immunofluorescence staining for nuclear visualization. Scale bar: 20 μm. (E) Fluorescence was quantified using ImageJ software. (F) Cellular senescence in TNFα-treated NPCs was assessed through β-gal staining. Scale bar: 50 μm. (G) Quantitative analysis of the percentage of senescent NPCs expressing β-gal in (F). (H) The protein expression levels of senescence markers (MMP3, p21 and p16) were assessed. ***P<0.001.

The impact of MA on the ECM metabolism of NPCs exposed to TNFα-induced inflammation was investigated. The results from qPCR and western blot analysis showed that TNFα downregulated the expressions of COL2A1 and ACAN, but MA helped alleviate this negative effect and inhibited TNFα-induced catabolism markers of the ECM, such as ADAMST5, ADAMTS4, MMP13 and MMP9 ( Figure 1C‒I). Moreover, immunofluorescence staining revealed that MA enhanced the expressions of COL2A1 and ACAN in an inflammatory microenvironment while suppressing the expressions of ADAMTS4 and MMP13 ( Figure 2A‒E).

### MA ameliorates TNFα-induced senescence in NPCs

Previous studies have shown that TNFα can enhance SA-β-gal activity and expedite senescence markers such as p21 and p16 [7]. Our results demonstrated that TNFα increased the activity of SA-β-gal and upregulated the expressions of senescence markers. In contrast, MA counteracts these effects ( Figure 2F‒G). Furthermore, the results also demonstrated that TNFα can lead to the upregulation of senescence-associated secretory phenotype markers, including MMP3, p21 and p16. However, MA inhibited the expressions of these markers under inflammatory conditions ( Figure 2H). These findings suggested that MA attenuates TNFα-induced NPC senescence.

### MA inhibits the PI3K/AKT/NF-κB signaling pathway in TNFα-stimulated NPCs

**Figure 3:**
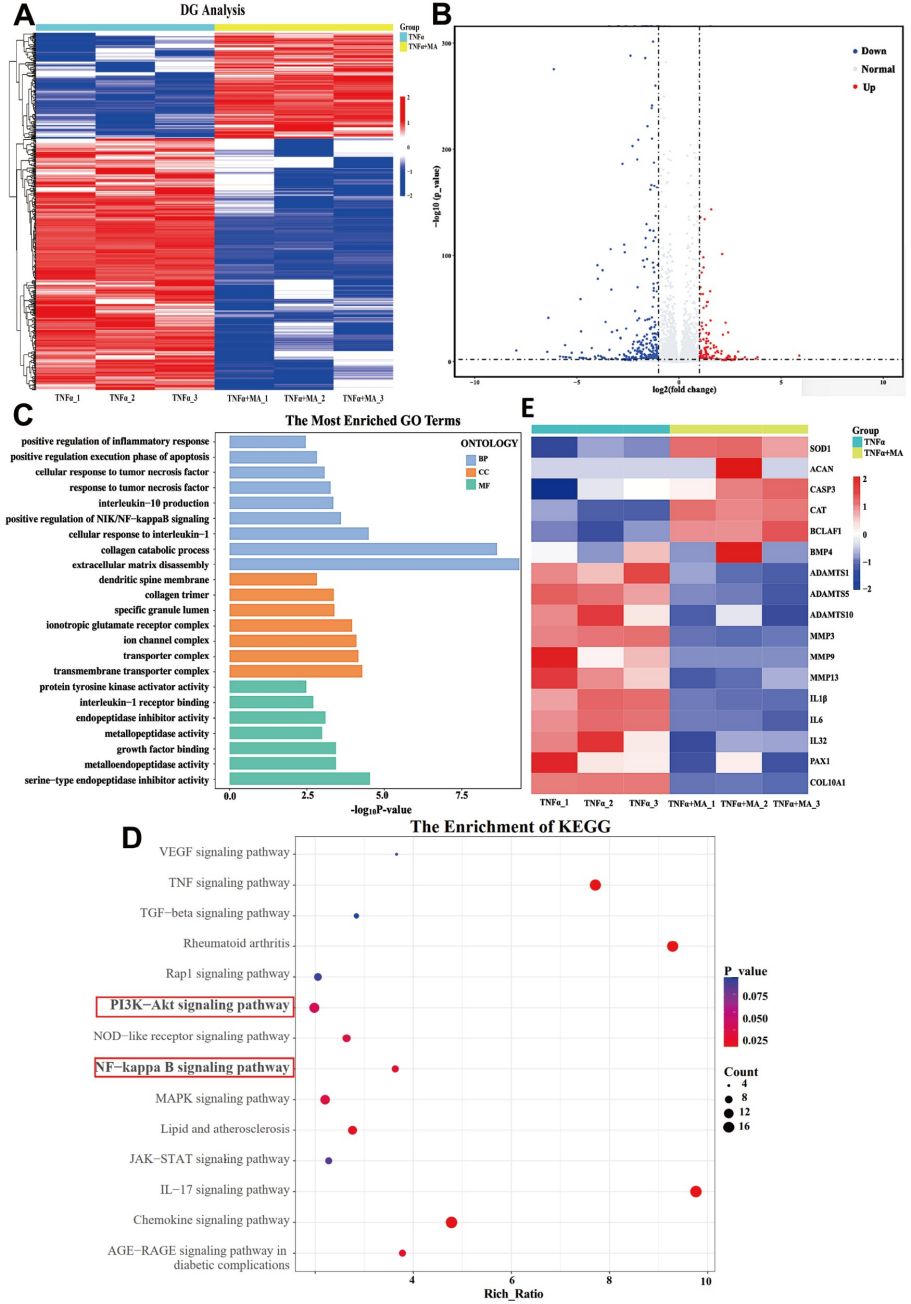
Figure 3 MA modulated the activation of the NF-κB and PI3K/AKT signaling pathways in TNFα-stimulated NPCs (A) Heatmap displaying the DEGs identified by RNA-seq in NPCs treated with TNFα alone or in combination with MA. (B) Map of the gene distribution in relation to volcanic activity. (C) The GO analysis terms, which included three analyses: BP (biological process), CC (cellular component), and MF (molecular function). (D) KEGG pathway enrichment analysis revealed pathways. (E) Heatmap displaying the expression levels of genes, including ACAN, MMPs, ADAMTSs, and inflammatory factors.

To further elucidate the mechanism of MA, RNA-seq analysis was conducted on human NPCs that were treated with either TNFα or TNFα plus MA. Our analysis revealed that 510 genes were differentially expressed (fold change >2 and adjusted *P* value <0.05), 151 genes were upregulated and 359 genes were downregulated following MA treatment ( Figure 3A,B). The results from the GO analysis suggested that MA treatment affected the positive regulation of the inflammatory response, ECM disassembly and collagen catabolic process ( Figure 3C). KEGG analysis demonstrated that MA treatment modulated the NF-κB and PI3K/AKT pathways ( Figure 3D). Notably, the mRNA expression levels of *MMPs* ( *MMP3*, *MMP9* and *MMP13*), *ADAMTSs* ( *ADAMTS1*, *ADAMTS5* and *ADAMTS10*) and inflammatory factors ( *IL-1β*, *IL-6* and *IL-32*) were downregulated in the MA-treated group, while the levels of SOD1, catalase and BCLAF1 were upregulated ( Figure 3E). These findings imply that MA has an impact on the diverse biological processes that are regulated by TNFα.

**Figure 4:**
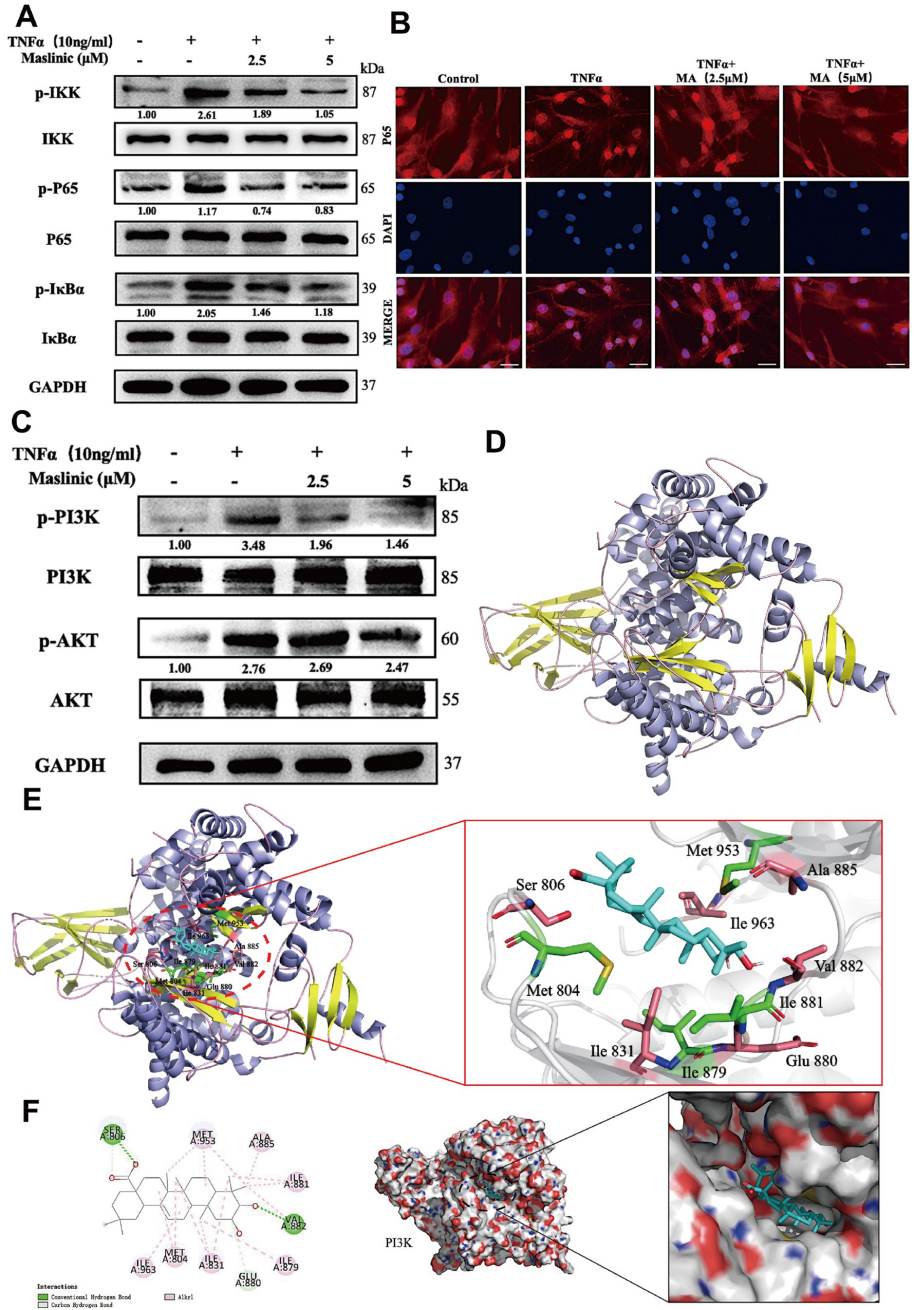
Figure 4 MA treatment modulated the activation of the NF-κB and PI3K/AKT signaling pathways in TNFα-stimulated NPCs (A) Western blot analysis of the NF-κB pathway in TNFα-treated NPCs with or without MA. (B) Immunofluorescence was utilized to detect p65. Scale bar: 20 μm. (C) Western blot analysis of the PI3K pathway in TNFα-treated NPCs treated with or without MA. (D) Ribbon model depicting the structure of PI3K. (E,F) The space-filling model illustrates the interaction between MA and PI3K, which results in a reaction with an interaction energy of –10.35 kcal mol−1.

Next, inactivation of the PI3K/AKT/NF-κB signaling pathway in NPCs treated with TNFα was investigated by MA treatment. Western blot analysis indicated that TNFα treatment significantly increased the ratios of p-IκBα, p-IKK and p-P65. However, these effects were mitigated by MA administration ( Figure 4A). Immunofluorescence microscopy showed that TNFα treatment caused p65 to translocate from the cytoplasm to the nucleus in NPCs. This movement was effectively suppressed by MA administration ( Figure 4B). Therefore, MA likely exerts an inhibitory effect on the activation of the NF-κB pathway.

To further validate the upstream signaling pathway of NF-κB, the effects of the PI3K/AKT signaling cascade in the group of NPCs treated with TNFα were investigated based on previous RNA-seq findings. Western blot analysis showed that TNFα enhanced the phosphorylation of PI3K and AKT in NPCs, but treatment with MA significantly attenuated this change ( Figure 4C). These findings suggested that MA treatment can effectively suppress the activation of the PI3K/AKT pathway.

Molecular docking was performed to analyze interactions between MA and PI3K. Table 3 presents the top 10 most favorable interactions and their corresponding interaction energies. Figures 1A and 4D depict the structures of both MA and PI3K. Based on the interaction energy in the ″pocket″ (‒10.35 kcal mol ^−1^), a strong connection was identified between MA and PI3K. Further analysis via the 2-D binding model revealed that certain amino acid residues of PI3K, including MET804, ILE831, ILE879, ILE881, VAL882, VAL885, MET953, ILE963, SER806 and GLU880, were implicated in van der Waals interactions with MA ( Figure 4E,F). These findings suggest that MA can directly interact with PI3K.

**Table:** **Table 3** Affinity between MA and PI3K in the top 10 models obtained from molecular docking

Mode	Affinity (kcal mol ^−1^)
1	‒10.35
2	‒10.31
3	‒9.39
4	‒9.35
5	‒9.34
6	‒9.34
7	‒9.34
8	‒9.34
9	‒9.33
10	‒9.32

### The *in vitro* effects of MA on IDD are compromised by the AKT agonist Sc-79

**Figure 5:**
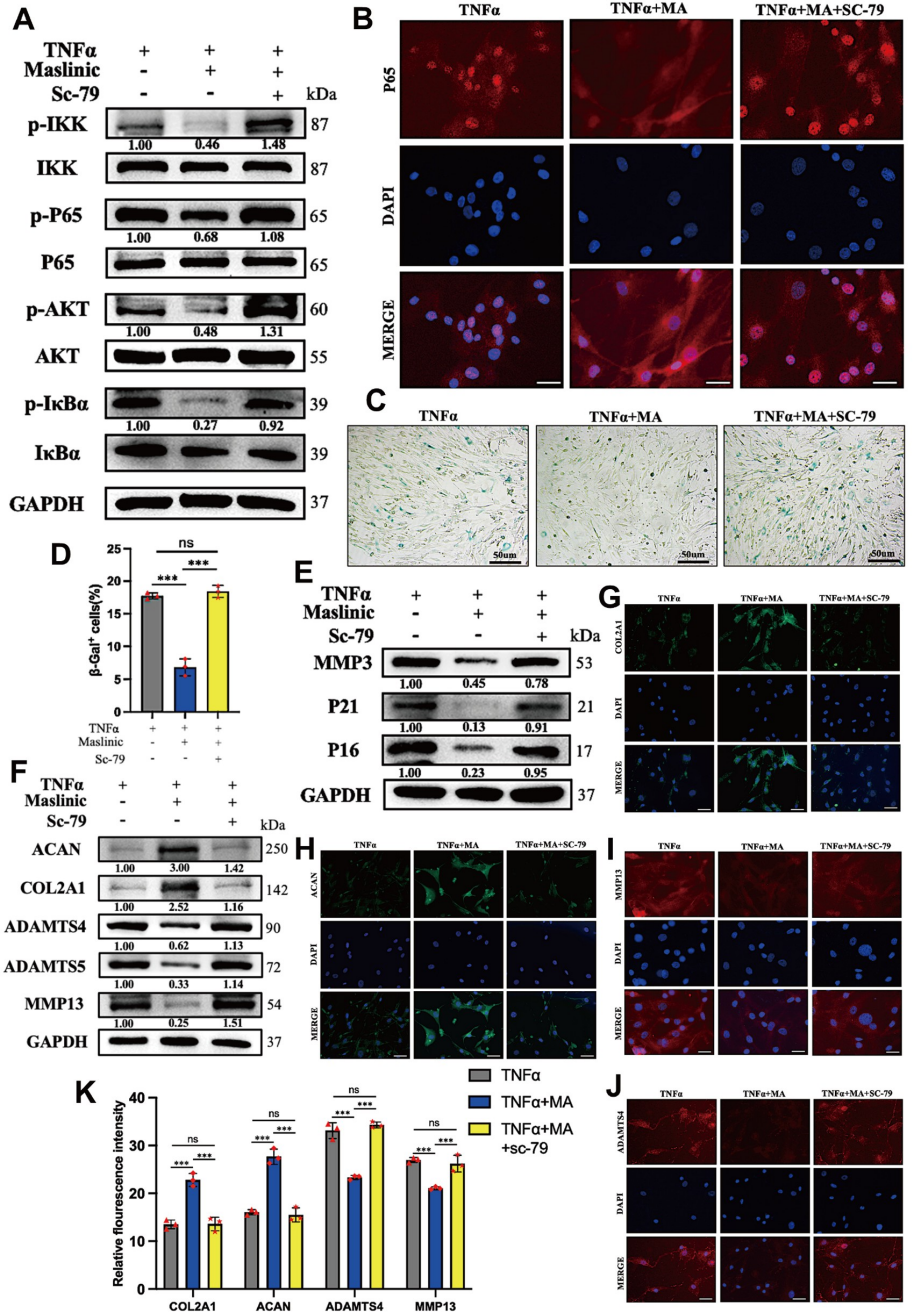
Figure 5 The AKT agonist Sc-79 effectively counteracted the inhibitory effects of MA on the PI3K/AKT/NF-κB signaling pathway (A) Western blot analysis of the PI3K/AKT/NF-κB pathway was performed after 30 min of treatment, as indicated. (B) Immunofluorescence staining was performed to determine the nuclear translocation of NF-κB p65. Scale bar: 20 μm. (C) Cellular senescence was assessed through β-gal staining. Scale bar: 50 μm. (D) Quantitative analysis of the percentage of senescent NPCs. (E) Western blot analysis was performed to detect senescence markers (MMP3, p21 and p16). (F) The protein expression levels of ACAN, COL2A1, ADAMTS4, ADAMTS5 and MMP13 were assessed via western blot analysis. (G–J) Immunofluorescence staining was utilized to detect the expressions of COL2A1, ACACN, MMP13 and ADAMTS4. Scale bar: 20 μm. (K) Fluorescence intensity was quantified using ImageJ. ***P<0.001.

To confirm the effect of MA on the PI3K/AKT/NF-κB pathway, NPCs were treated with Sc-79, an AKT agonist. Our findings showed that Sc-79 attenuated the suppressive effect of MA on the PI3K/AKT/NF-κB signaling pathway ( Figure 5A) and weakened the inhibitory effect of MA on NF-κB p65 nuclear translocation ( Figure 5B). Compared to those in the MA treatment group, the SA-β-Gal activity in the Sc-79 treatment group was greater ( Figure 5C,D), and the senescence marker levels were significantly elevated ( Figure 5E). Furthermore, western blot analysis revealed significant upregulation of catabolism markers and significant downregulation of anabolism markers in response to Sc-79 treatment ( Figure 5F). Finally, the results were visually confirmed by immunofluorescence staining ( Figure 5G‒K).

### MA ameliorates the development of IDD *in vivo*

**Figure 6:**
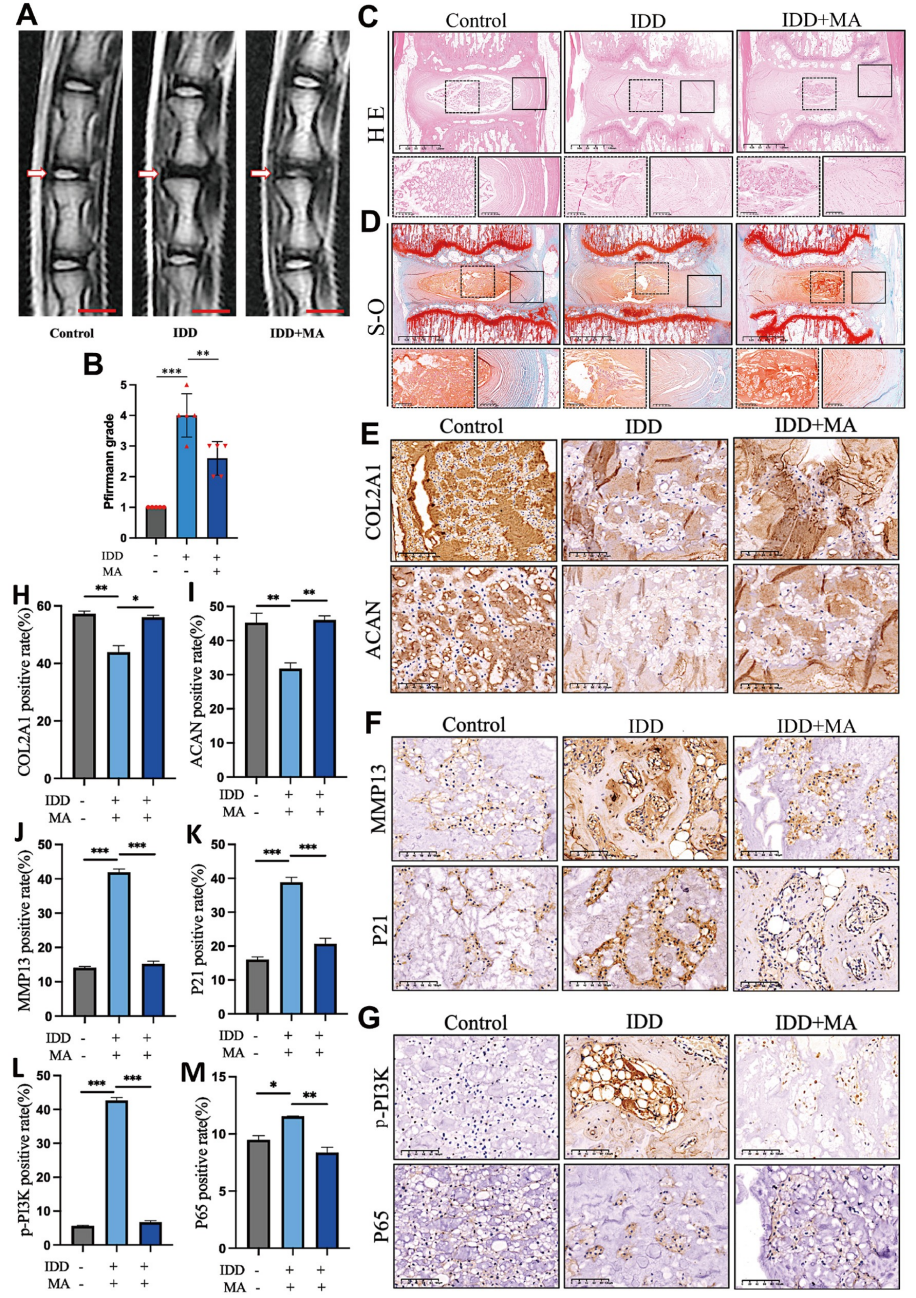
Figure 6 MA mitigates IDD progression in a rat model (A) MRI of rat tails with corresponding treatments. Scale bar: 5 mm. (B) Analysis of Pfirrmann scores from images across multiple groups (n=5). (C,D) Illustrations depicting H&E staining and cross-SO staining of samples from various experimental groups. (E–G) Immunohistochemical staining was performed to detect the expressions of COL2A1, ACAN, MMP13, p21, p-PI3K and NF-κB p65 in rat intervertebral discs. Scale bar: 100 μm. (H–M) Statistical analysis of the immunohistochemical staining results. *P<0.05, **P<0.01, ***P<0.001.

To study the protective effect of MA on IDD progression *in vivo*, we established a surgical acupuncture-induced rat model. The rats were given either saline or MA (100 mg/kg) orally once a day for four weeks. Destonal degeneration scores were evaluated using the Pfirrmann grading system based on MRI [28]. The Pfirrmann grade was greater in the IDD group than in the control group. The IDD+MA group exhibited a reduction in Pfirrmann’s grade score ( Figure 6A,B). Further examination using HE and SO staining revealed that MA improved the disordered lamellae and disrupted the borders ( Figure 6C,D).

Immunohistochemical staining revealed that IDD led to the downregulation of anabolic ECM markers such as COL2A1 and ACAN, but MA was found to mitigate this effect ( Figure 6E). Furthermore, MMP13 and p21 expressions were upregulated in the IDD group, but MA suppressed their expressions ( Figure 6F). Finally, our findings suggest that MA inhibits p-PI3K expression and reverses the nuclear translocation of p65. ( Figure 6G). The statistical results are shown in Figure 6H‒M. In brief, MA exhibits therapeutic potential *in vivo* for mitigating IDD progression due to its suppressive effects on the PI3K/AKT/NF-κB pathways.

### ECM metabolism disorders and activation of the PI3K/AKT pathway in degenerative nucleus pulposus tissues

Immunohistochemical analysis revealed significantly lower levels of the anabolism markers COL2A1 and ACAN in the degenerative nucleus pulposus group than in the control group. The expression levels of MMP13, MMP9, p16 and TNFα were greater in the degenerative group ( Supplementary Figure S1A,B). Additionally, immunohistochemical analysis revealed that the PI3K/AKT signaling pathway was activated in IDD tissues ( Supplementary Figure S1C). The statistical results are shown in Supplementary Figure S1D‒J.

## Discussion

Previous studies have demonstrated that IDD is the primary etiology of LBP [1]. However, the quest for novel and efficacious drugs for treating IDD is a formidable challenge. MA, which is found in olive *(Olea europaea)*, has been reported to have anti-inflammatory, antioxidant and antitumor properties [ 21, 30, 31]. Studies have revealed that MA exhibits superior antioxidant properties to those of other MA species due to its additional hydroxyl groups [32]. Furthermore, the absence of adverse effects in hematological, clinical biochemical and histopathological evaluations implies a high safety margin for orally administered MA [33]. We confirmed the effectiveness of MA in relieving IDD *in vivo* by oral administration to model rats. There was no death or abnormal performance unrelated to the model in rats, which also verified the safety of MA and provided a guarantee for the preparation of MA oral drugs.

It has been reported that MA can protect the skeletal system by preventing osteoarthritis and reducing bone loss [ 25, 34]. Studies have also shown that MA can improve muscle mass and alleviate knee joint pain in patients with knee osteoarthritis [ 22, 35]. These findings imply that MA could mitigate IDD. Inhibiting inflammatory cytokines such as TNFα can mitigate the progression of IDD [ 5, 6, 36]. Therefore, TNFα was used to simulate an inflammatory microenvironment in subsequent experiments. The safe concentrations of MA in NPCs were first determined, followed by observation of how MA protects against ECM metabolism disorders while delaying IDD progression in a rat model. Our research showed that MA treatment significantly inhibited the expressions of TNFα-induced proinflammatory factors, downregulated matrix metalloproteinase levels and upregulated matrix component levels in intervertebral discs. Thus, MA plays a significant role in balancing anabolism and catabolism in the ECM of NPCs, which suppresses the senescence-associated secreted phenotype.

The NF-κB pathway, which serves as a downstream signaling pathway of the TNFα-mediated inflammatory environment, is crucial for the progression of IDD [37]. The NF-κB pathway is located downstream of the PI3K/AKT pathway in NPCs [14]. Through RNA-seq, we found that MA regulates the PI3K/AKT and NF-κB signaling pathways and verified this finding experimentally, which is consistent with previous research results, demonstrating the potential therapeutic application of MA in managing diseases associated with aberrant PI3K/AKT/NF-κB activation [ 30, 31, 34].

In addition, MA treatment significantly upregulated the expressions of SOD1 and CAT in NPC cells, indicating that MA is helpful for resisting oxidative stress and responding to the antioxidant and anti-inflammatory effects of MA mentioned above. MA also significantly upregulated CASP3 and BCLAF1 expressions. Caspase-3, encoded by *CASP3*, is an important molecule involved in classical apoptosis, and BCLAF1 recruits cells to damage sites and promotes apoptosis after DNA damage [38], which seems to contradict the inhibitory effect of MA on NPC ECM dysfunction. However, the proapoptotic effect of BCLAF1 is still controversial because there is no obvious difference in apoptosis in BCLAF1-deficient mice, and BCLAF1 can promote the proliferation of colorectal cancer cells [39]. However, the role of MA-induced CASP3 and BCLAF1 in TNFα-induced IDD needs further study. MA can reportedly inhibit inflammation and apoptosis in acute kidney injury by inhibiting the activation of MAPK signaling [40]. However, another study showed that the inhibitory effect of MA on the MAPK pathway promotes apoptosis [41]. Further studies of the contribution of the MAPK signaling pathway detected by our sequencing in IDD are needed to clarify how MA regulates MAPK signaling.

Molecular docking methods are valuable in the field of drug discovery because they can reduce costs and simplify the process of screening novel compounds by identifying potential targets. With this technology, we found that MA could directly bind to the pocket of PI3K, indicating its potential interaction with PI3K and subsequent suppression of its phosphorylation during TNFα-induced inflammation.

In conclusion, our findings demonstrate that MA not only affects IDD *in vitro* but also *in vivo* by inhibiting TNFα-induced inflammation while alleviating ECM homeostasis disorder and senescence in NPCs. Therefore, MA may induce conservative treatment and rehabilitation treatment in LBP patients with IDD. However, further studies are necessary to evaluate the interaction between MA and PI3K using fluorescence- or biotin-labelling techniques *in vivo*.

## Supporting information

23537supplementary_Figure_S1

## Supplementary Data

Supplementary data is available at *Acta Biochimica et Biophysica Sinica* online.
