# Supplementary material for: Maslinic acid alleviates intervertebral disc degeneration by inhibiting the PI3K/AKT and NF-κB signaling pathways: Role of MA in alleviating intervertebral disc degeneration
Source: Acta Biochim Biophys Sin (Shanghai). 2024 Mar 18;56(5):776–88. doi: 10.3724/abbs.2024027 (PMC11187486; doi:10.3724/abbs.2024027)
Supplement: 23537supplementary_Figure_S1 [file 23537supplementary_Figure_S1.pdf]

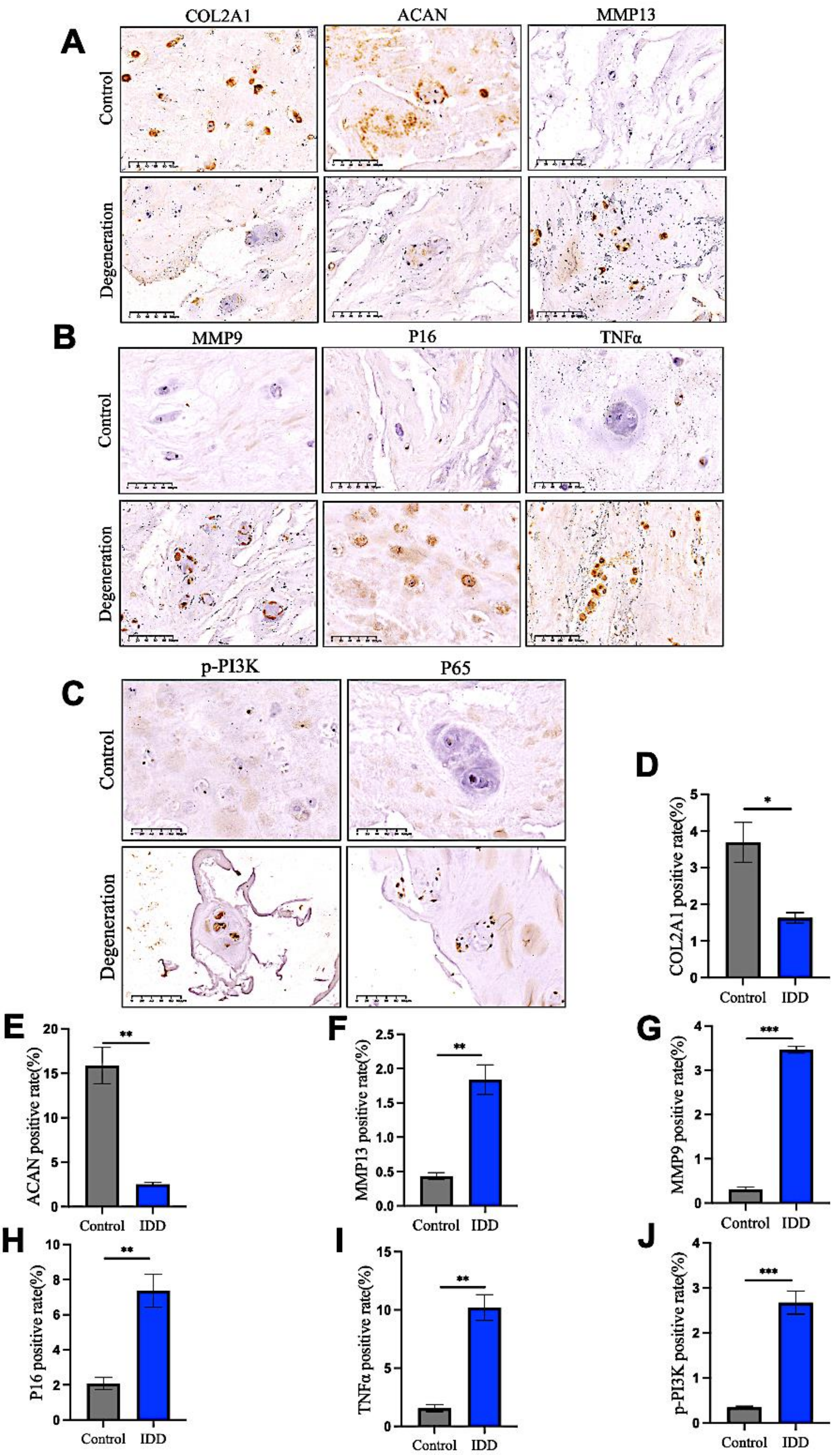
A
COL2A1
ACAN
MMP13
Control
Degeneration
B
MMP9
P16
TNFα
Control
Degeneration
C
p-PI3K
P65
Control
Degeneration
D
COL2A1 positive rate(%)
Control
IDD
E
ACAN positive rate(%)
Control
IDD
F
MMP13 positive rate(%)
Control
IDD
G
MMP9 positive rate(%)
Control
IDD
H
P16 positive rate(%)
Control
IDD
I
TNFα positive rate(%)
Control
IDD
J
p-PI3K positive rate(%)
Control
IDD

Supplementary Figure S1. The ECM homeostasis disorder and the activation of PI3K/AKT pathway in degenerative NP tissues (A–C) Immunohistochemical staining was performed for COL2A1, ACAN, MMP13, MMP9, p16, TNFα, p-PI3K and P65 in clinical NP tissues with varying degrees of degeneration. Scale bar: 100 μm. (D–J) Statistical analysis of immunohistochemical staining results. *P<0.05, **P< 0.01, and ***P<0.001.
